# Phenylalanine-Free Infant Formula in Patients with Phenylketonuria: A Retrospective Study

**DOI:** 10.3390/nu16142204

**Published:** 2024-07-10

**Authors:** Ozlem Yilmaz Nas, Catherine Ashmore, Sharon Evans, Alex Pinto, Anne Daly, Nurcan Yabancı Ayhan, Anita MacDonald

**Affiliations:** 1 Birmingham Children’s Hospital, Birmingham B4 6NH, UK; catherine.ashmore@nhs.net (C.A.); evanss21@me.com (S.E.); alex.pinto@nhs.net (A.P.); a.daly3@nhs.net (A.D.); anita.macdonald@nhs.net (A.M.); 2 Department of Nutrition and Dietetics, Ankara Yildirim Beyazit University, Ankara 06760, Turkey; 3 Department of Nutrition and Dietetics, Ankara University, Ankara 06760, Turkey; nyabanci@ankara.edu.tr

**Keywords:** phenylketonuria, infancy, infant protein substitute, growth, metabolic control

## Abstract

The long-term efficacy and use of phenylalanine-free infant amino acid formula (PFIF) is understudied. This retrospective, longitudinal study evaluated PFIF (PKU Start: Vitaflo International) in children with phenylketonuria, collecting data on metabolic control, growth, dietary intake, and symptoms and the child’s experience with PFIF. Twenty-five children (12 males, 48%) with a median age of 3.6 years (2.0–6.2 years) were included. During 24 months follow-up, children maintained normal growth and satisfactory metabolic control. The protein intake from protein substitutes increased from 2.7 at 6 months to 2.8 g/kg/day at 24 months, while natural protein decreased from 0.6 to 0.4 g/kg/day. By 24 months, most children (n = 16, 64%) had stopped PFIF, while nine (36%) continued with a median intake of 450 mL/day (Q1:300 mL, Q3: 560 mL). Children who continued PFIF after 24 months of age had higher energy and fat intakes with higher weight/BMI z-scores compared with those who stopped earlier (*p* < 0.05). Constipation was reported in 44% of infants but improved with age. Initial difficulty with PFIF acceptance was reported in 20% of infants but also improved with time. Prolonged use of PFIF in pre-school children may contribute to poor feeding patterns and overweight; thus, replacing the majority of the protein equivalent provided by PFIF with a weaning protein substitute by 12 months and discontinuing PFIF before 2 years is recommended.

## 1. Introduction

Phenylketonuria (PKU, OMIM 261600) is an inherited autosomal recessive disorder of amino acid metabolism caused by a deficiency of phenylalanine hydroxylase (PAH). This enzyme together with its essential cofactor, tetrahydrobiopterin, converts the amino acid phenylalanine into tyrosine [1]. Without the functional PAH enzyme, phenylalanine accumulates in the blood, tissues, and brain and if untreated, causes severe neurological impairment with intellectual disability [1,2,3]. The global prevalence of PKU is 1 in 23,930 live births, ranging from 1 in 4500 in Italy to 1 in 125,000 in Japan, with the prevalence in the UK being 1 in 10,000 [4]. Early detection through newborn screening is essential to safeguard a healthy outcome. Any infant with a blood phenylalanine level above 360 µmol/L should commence a phenylalanine restrictive diet immediately, preferably within the first 10 days of life [1].

Dietary management in infancy comprises the administration of a phenylalanine-free infant amino acid formula (PFIF) together with controlled but limited amounts of breastmilk or standard infant formula that will provide essential phenylalanine requirements [1]. In PKU, phenylalanine requirements start at around 55 mg/kg/day in infants aged 0–3 months, decreasing to 27 mg/kg/day by 12 months, and children with classic PKU may only tolerate 200–500 mg/day of phenylalanine. However, these requirements are influenced by many factors including the severity of the disorder, energy intake, and the dosage and distribution of PFIF intake [1].

The infant’s first year is a critical time of rapid growth and development, supported by a high rate of protein synthesis. The nutritional composition of PFIF is comparable to human milk. It contains macro and micronutrients, electrolytes, and trace elements, but is based on phenylalanine-free amino acids, and the amounts of non-phenylalanine essential and conditionally essential amino acids should at least be comparable to human milk [5]. It contains long-chain fatty acids (arachidonic and docosahexaenoic) and may contain novel ingredients such as prebiotics. The osmolarity of PFIF is similar to breastmilk [6]. PFIF must comply with the relevant worldwide regulations to ensure its safety and quality, and all raw materials used must be approved for infants [7,8]. Its nutritional composition should ensure appropriate growth, optimal development, and promote healthy metabolic programming [6], be readily digestible, and well tolerated. The production of PFIF requires rigorous chemical processes and sophisticated purification techniques, affecting the cost of the end product [9,10,11,12,13].

Although PFIF has evolved over the years [14,15], there are limited data on its efficacy and safety, particularly regarding long-term outcomes. We recently published a longitudinal, prospective, open, multi-center study [16] evaluating the acceptability, tolerability, growth, and metabolic control of a PFIF in infants with PKU over a 12-month duration. The PFIF was well tolerated, infant growth was normal, and good metabolic control was maintained, but only a small number of subjects were studied. It was concluded that further research was necessary, investigating a greater number of infants. This current study addresses the gap in long-term safety and efficacy data for PFIF by evaluating a larger cohort of infants over an extended period, contributing to the current literature on PFIF. Therefore, in this retrospective study, we evaluated the growth, metabolic control, and dietary intake of a larger group of infants with PKU who had been given a PFIF and treated in one PKU center from 2017 to 2023.

## 2. Materials and Methods

### 2.1. Project Design

**Figure 1:**
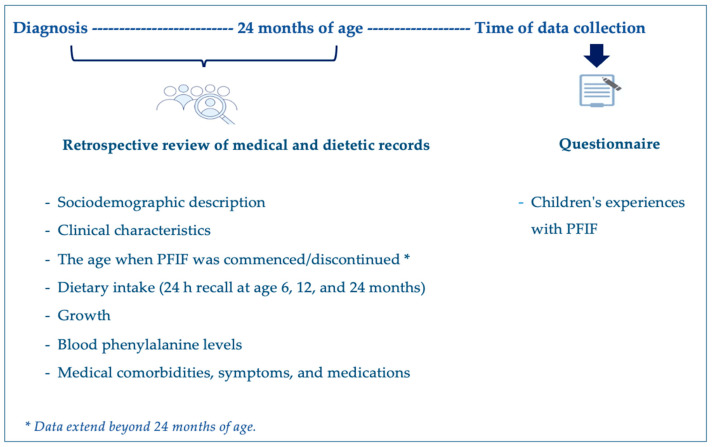
Schematic diagram of the study design.

We conducted a single-center, retrospective study of children with PKU who were prescribed a PFIF (PKU Start: Vitaflo, Liverpool UK; Nestlé Health Science, Vevey, Switzerland) from diagnosis. The study design is illustrated in Figure 1. The eligibility criteria included a diagnosis of PKU via newborn screening, and management with a phenylalanine-restricted diet supplemented with a PFIF. Infants were excluded if they were aged <1 year at the time of data collection, diagnosed with hyperphenylalaninemia (blood phenylalanine concentrations < 360 µmol/L when untreated), or had comorbidities that altered dietary management (e.g., diabetes).

### 2.2. Data Collection

Medical and dietetic records were accessed and the following retrospective information was collected from the time of diagnosis until the child’s second birthday: demographic characteristics (e.g., age, sex, ethnic origin, number of siblings, family size, marital status, and maternal education), PKU classification based on infant genetic mutations or pre-treatment blood phenylalanine levels (classical PKU [>1200 µmol/L], moderate PKU [600–1200 µmol/L], or mild PKU [360–600 µmol/L], blood phenylalanine levels, nutritional intake (at 6, 12, and 24 months of age), anthropometry, medical comorbidities, medications, and any viral infections or symptoms such as vomiting, diarrhea, constipation, and respiratory symptoms that had been documented. The age at which PFIF was commenced and discontinued, extending beyond the child’s second birthday, was also recorded for comprehensive analysis.

Parents completed a six-item open-ended, non-validated questionnaire that explored the individual experiences with PFIF, including the age at which night feeds were discontinued and the maximum daily volume of the PFIF consumed. Parents described any infant behavioral feeding issues or difficulties with the PFIF. Data were collected on the type of low protein milk or fluids post PFIF use.

### 2.3. Blood Phenylalanine Control

Weekly or twice-weekly morning fasting heel prick blood spots for phenylalanine were collected on filter cards (Perkin Elmer 226, UK Standard NBS) by family caregivers at home. All caregivers received blood spot training from a specialist nurse. The blood spot samples were sent via first-class mail to the hospital laboratory. The filter cards had a standardized thickness, and blood phenylalanine was calculated from a 3.2 mm punch using MS/MS tandem mass spectrometry. Good metabolic control was defined as the maintenance of blood phenylalanine levels within the therapeutic target range of 120 to 360 µmol/L [17].

### 2.4. Anthropometry

Weight and length was measured by an experienced healthcare professional, either in a home setting or in hospital/community clinics. Length was measured using a Holtain Harpenden infantometer (Holtain Ltd., Crymych, UK) and weight on calibrated digital scales (Seca, Medical Measuring Systems and Scales, Model 875, Birmingham, UK). Weight was measured to the nearest 0.1 g and length to the nearest 0.1 cm. All weight and length measurements were recorded in patient-held records and subsequently converted into age and sex-specific z-scores for weight, length, and body mass index (BMI) using the WHO Anthro software version 1.0.3 [18,19].

### 2.5. Dietary Assessment

Pediatric dietitians documented 24 h diet histories by a recall method either in clinics, during home visits or in virtual clinic reviews. They were analyzed at age 6 (±1 month), 12 (±1 month), and 24 months (±1 month). Energy, macronutrients, and protein equivalent (combined intake from PFIF and weaning PS) intake were evaluated using the Nutritics program [20]. Infants were excluded from dietary analyses if they were receiving breastmilk at analysis time points (e.g., 6 months). The percentage of estimated average requirements (EAR%) for energy was calculated by comparing the energy intake with the age- and sex-specific EARs from the UK Scientific Advisory Committee on Nutrition/Committee on Medical Aspects of Food Policy reports [21].

### 2.6. Study Formula

Table 1 shows the nutritional composition of the study PFIF compared to a standard infant formula. The study PFIF (PKU Start: Vitaflo, Liverpool, UK, Nestlé Health Science, Vevey, Switzerland) contained essential and non-essential amino acids, carbohydrates, fat, vitamins, minerals, trace elements, arachidonic acid, and docosahexaenoic acid. It was reconstituted by adding 4.7 g (1 scoop) of powder to 30 mL of water and provided 2 g/100 mL of protein equivalent at a standard dilution of 14.1% and an osmolality of 350 mOsm/kg. The nutritional composition of the PFIF complied with the Commission Delegated Regulation (EU) (2016/128), which supplements the Regulation (EU) No 609/2013 of the European Parliament and of the Council [7]. The study PFIF was provided on medical prescription, free of charge via the UK National Health Service.

### 2.7. Statistical Analysis

The statistical analysis was performed using Microsoft Excel (version 16.71) [22] and SPSS^®^ software (version 26) [23]. Descriptive statistics were reported as medians, first quartile (Q1), and third quartile (Q3), or as ranges for continuous variables, and as percentages for categorical variables. The weight, length, and BMI-z-scores and blood phenylalanine levels were analyzed after categorizing them into three age groups: 0–6 months, 7–12 months, and 13–24 months. Independent sample *t*-tests were performed to assess the differences in weight, length, and BMI z-scores by sex and PFIF discontinuation (before vs. after 24 months), in addition to the differences in blood phenylalanine, dietary intakes, and PFIF discontinuation (before vs. after 24 months) at defined time points. The associations between multiple independent social variables (sex, number of siblings, family size, maternal education, parental marital status, and living conditions) and PFIF discontinuation (before vs. after 24 months) were assessed using the chi-square test. Statistical significance was defined as a *p*-value of <0.05.

No sample size calculations were performed since the data for all eligible patients who were diagnosed with PKU and prescribed PFIF at Birmingham Children’s Hospital during the study period were included in the study.

### 2.8. Ethical Approval

The study received a favorable ethical opinion from the Wales Research Ethics Committee with reference number 23/WA/0143 and IRAS (Integrated Research Application System) ID 325952. The study was conducted in accordance with the ethical principles of the Declaration of Helsinki and UK law and Good Clinical Practice guidelines, and UK Health Research Authority (HRA) approval and local NHS R&D/site approval was obtained. Written informed consent was obtained from at least one caregiver with parental responsibility for all subjects.

## 3. Results

### 3.1. Subjects

The study evaluated the medical and dietetic records of 25 children (n = 12 male, 48%) who were diagnosed with PKU, and all were prescribed the same PFIF. Most of the children were European/Caucasian origin (n = 22, 88%), two (8%) were Pakistani Asian origin, and one (4%) was mixed race. At the time of the study evaluation, the median age of the children was 3.6 years (range: 2.0 to 6.2 years). The majority (n = 21, 84%) had classical PKU, three (12%) had moderate PKU, and one (4%) had mild PKU.

### 3.2. Introduction of PFIF and Transition to Weaning Protein Substitute and Solid Foods

Following diagnosis, all infants (n = 25) received the PFIF in combination with breastmilk or standard infant formula. Treatment commencement was usually by the age of 10 days (n = 19/25, 76%). Eight infants (32%) received breastmilk as their primary source of phenylalanine for a median duration of 5.3 months (range: 2.0 to 11.0 months), while 16 infants (64%) received standard infant formula. One infant (4%) was prescribed extensively hydrolyzed formula due to cow’s milk allergy.

The median age of introducing weaning solid foods was 18 weeks (range: 15 to 24 weeks). Around the same time, all children were introduced to a weaning semi-solid protein substitute, starting with 5 g/day of powder (providing 2 g/day of protein equivalent), given once or twice daily and mixed with 5 mL of water to a paste-like consistency. When the semi-solid weaning protein substitute was introduced, the aim was to reduce the volume of PFIF, with breastfeeds or standard infant formula being gradually replaced by weaning foods containing an equivalent phenylalanine content. Of the 25 children, 16 (64%) discontinued PFIF by 2 years of age (median age: 1.5 years [range: 1.1 to 1.9 years]). In the remaining 9 children (36%), 6 (24%) discontinued the PFIF at a median age of 2.3 years (range: 2.1 to 3.0 years), and at the time of this review, 3 children (aged 3.6, 5.1, and 6.2 years) still consumed PFIF as a social drink due to parental or child preference and rejection of alternative protein-free milks.

### 3.3. Metabolic Control

**Figure 2:**
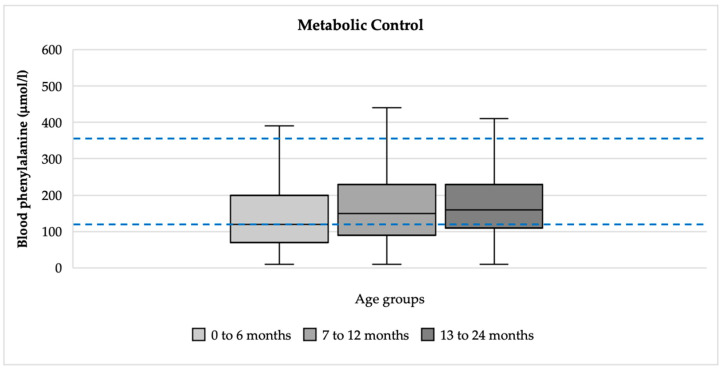
Blood phenylalanine control by age groups. Blue lines represent the target therapeutic range of 120–360 µmol/L.

A total of 3497 blood phenylalanine levels were analyzed for children (n = 25) aged between 0 and 2 years. The median blood phenylalanine levels were within the target therapeutic range of 120–360 µmol/L [17]: aged 0 to 6 months: 120 µmol/L (Q1: 70 µmol/L, Q3: 200 µmol/L); aged 7 to 12 months: 150 µmol/L (Q1: 90 µmol/L, Q3: 230 µmol/L); and aged 13 to 24 months: 160 µmol/L (Q1: 110 µmol/L, Q3: 230 µmol/L) (Figure 2).

The percentage of children with blood phenylalanine levels that exceeded the upper target range of 360 µmol/L at some point in time was 10% or less. The percentage for each age group was 6% at 0 to 6 months, 8% at 7 to 12 months, and 10% at 13 to 24 months.

### 3.4. Anthropometric Changes

**Figure 3:**
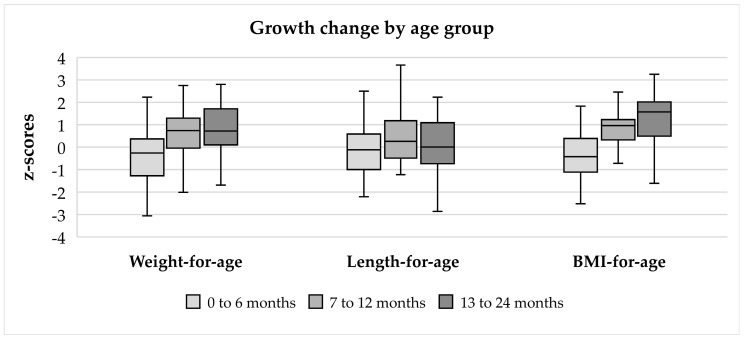
Changes in median weight-for-age, length-for-age, and BMI-for-age z-scores by age groups.

A total of 743 weight, length, and BMI-z-scores were analyzed. Table 2 and Figure 3 show the changes in the median weight-for-age, length-for-age, and BMI-for-age z-scores by age groups. Children had a median birth weight of 3.4 kg (Q1: 3.1 kg, Q3: 3.6 kg). Growth was within normal parameters, although BMI increased with age.

### 3.5. Dietary Intake

Table 3 presents the actual dietary intakes at 6 months (n = 21), 12 months (n = 24), and 24 months (n = 25) of age.

From 6 to 12 months of age, the median percentage of total energy and protein intake from PFIF decreased: total energy from 66% (Q1: 60%, Q3: 72%) to 40% (Q1: 31%, Q3: 50%), and the total protein from 57% (Q1: 48%, Q3: 66%) to 31% (Q1: 19%, Q3: 41%). By age 24 months, 16 children (64%) stopped using PFIF. Nine children (36%) had a median intake of 450 mL/day (Q1: 300 mL, Q3: 560 mL), contributing to 29% (Q1: 21%, Q3: 39%) and 23% (Q1: 16%, Q3: 31%) of their total energy and protein intake, respectively.

### 3.6. Metabolic Control—PFIF Discontinuation before vs. after 24 Months of Age

Children who discontinued PFIF after 24 months of age had significantly higher blood phenylalanine levels when aged 0 to 6 months (*p* < 0.05), but significantly lower (*p* < 0.05) blood phenylalanine levels when aged 13 to 24 months than those who discontinued PFIF before 24 months (Table 4). Sixteen per cent (n = 4) of children still received night feeds in the group that continued PFIF after 24 months of age.

### 3.7. Growth—Male vs. Female

No significant differences in length and BMI-for-age z-scores were observed between males and females in any age groups (*p* > 0.05). Females, however, had statistically significantly higher weight-for-age z-scores when aged 0 to 6 months and 13 to 24 months (*p* < 0.05) (Table 5).

### 3.8. Growth—PFIF Discontinuation before vs. after 24 Months of Age

There were no significant differences in the length-for-age z-scores between those who discontinued PFIF before or after 24 months of age (*p* > 0.05). However, children who discontinued PFIF after 24 months of age had significantly higher weight-for-age and BMI-for-age z-scores in the 13 to 24 months age group compared to those who discontinued before 24 months of age (Table 6).

### 3.9. Dietary Intake—PFIF Discontinuation before vs. after 24 Months of Age

At 6 months of age, there were no significant differences in energy and macronutrient intake between those who discontinued PFIF before or after 24 months of age. However, children who discontinued PFIF after 24 months of age had a significantly higher energy intake (kcal/day) and percentage estimated average requirement (EAR%) at 24 months, and fat intake (g/day and g/kg/day) at both 12 and 24 months compared to those who discontinued PFIF before 24 months of age (*p* < 0.05). No significant differences were observed in the total protein intake, natural protein intake, or protein equivalent intake from protein substitutes at any age between the two groups (*p* > 0.05) (Table 7).

### 3.10. Socioeconomic Factors—PFIF Discontinuation before vs. after 24 Months of Age

No significant associations were found between various social factors including sex, number of siblings, family size, and the timing of PFIF discontinuation, except for parental marital status (Table 8). A significantly higher percentage of children among single-parent families (71%, n = 5/7) continued PFIF after 24 months of age, compared to two-parent families (22%, n = 4/18). Children of mothers with lower education levels (left school at 16 years with no further education) were more likely to continue PFIF after 24 months of age (54%, n = 7/13) than mothers of children with higher education, e.g., diplomas/degrees (17%, n = 2/12).

### 3.11. Symptoms and Medications

**Figure 4:**
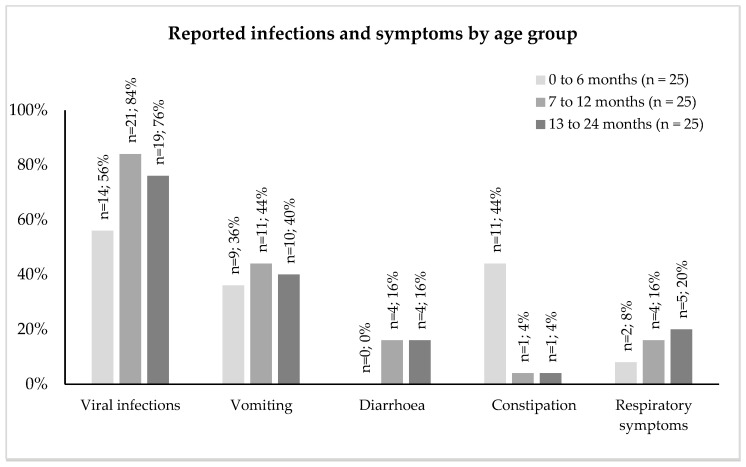
Viral infections and symptoms by age group.

Figure 4 describes any documented viral infections (e.g., varicella, COVID-19) and other symptoms including vomiting, diarrhea, constipation, and respiratory symptoms (dyspnea, coughing, and wheezing). Viral infections and vomiting were prevalent across all age groups, with vomiting usually associated with infections. Constipation was reported in 44% (n = 11) of infants aged 0 to 6 months but improved with age.

The use of prescribed medications, including laxatives, antacids, and others (anti-colic, proton pump inhibitors, and H2 blockers), decreased from age 0 to 6 months to 13 to 24 months: 20% (n = 5) to 4% (n = 1) for laxatives, 32% (n = 8) to 0% (n = 0) for antacids, and 12% (n = 3) to 4% (n = 1) for anti-colic, proton pump inhibitors, and H2 blockers.

### 3.12. Experience with the PFIF

The majority of mothers (n = 20, 80%) reported using the non-validated six-item questionnaire that their child accepted the formula without any issues, while five children (20%) initially experienced difficulty with acceptance but this improved with time. Five children (20%) had occasional reflux, but parents did not attribute this to the PFIF.

The majority of children (n = 14, 56%) stopped night feeding by one year of age. Throughout the feeding period with PFIF, the mothers reported a wide variation in PFIF intake, with a median highest volume of 840 mL/day. After discontinuing PFIF, children mainly consumed water (44%; n = 11), low-protein milk (36%; n = 9), and/or diluted fruit juice (32%; n = 8).

## 4. Discussion

All specialist infant formula should be systematically evaluated to ensure its safety, tolerance, and acceptance. Although our recent study [16] in infants with PKU provided preliminary evidence on these aspects of this PFIF, its limited duration and small sample size highlighted the need for further comprehensive evaluations in a larger cohort of infants. To address this gap, the present study provides long-term retrospective data on metabolic control, growth, dietary intake, symptoms, and experience of using a specialized formula in a cohort of 25 infants from diagnosis until 2 years of age.

Following diagnosis, all infants received PFIF and the majority tolerated it well. Even though some had initial difficulties with acceptance, this improved with age. Introduced from around 15 to 24 weeks, semi-solid weaning phenylalanine-free protein substitute gradually replaced PFIF. By the age of 2 years, the majority of children (n = 16, 64%) had stopped their PFIF intake. Nonetheless, 9 (36%) continued to receive PFIF after the age of 2 years, with a median daily intake of 450 mL at 2 years. This group (children who discontinued PFIF after 2 years of age [n = 9, 36%]) had a significantly higher energy intake, when expressed as a percentage of estimated average requirement (%EAR), and fat intake at 24 months of age, along with significantly higher weight and BMI-for-age z-scores from the age of 1 year onwards (*p* < 0.05), which was a concern. There were no significant differences in the length-for-age z-score and protein intake (natural and protein equivalent from protein substitutes) between the two groups. Children maintained satisfactory blood phenylalanine control, and growth remained within normal parameters throughout the study period.

The prolonged use of PFIF and delayed transition from bottle to cup has been previously reported in children with PKU [24]. Similarly, a group of children in this study (n = 9, 36%) continued to receive PFIF beyond 2 years of age, despite ongoing advice from dietitians to discontinue its use. MacDonald et al. [25] has recommended limiting PFIF intake (<600 mL/day) in later infancy and replacing it mainly with a weaning protein substitute by 12 months of age. Continuing bottle use later than the age of 18–24 months has been associated with a higher risk of overweight and obesity between 3 and 5 years of age [26,27]. Ensuring the timely discontinuation of PFIF is essential to prevent excessive energy and fat intake, help avoid feeding problems with solid foods, and prevent potential long-term growth issues. In addition, the gradual reduction and timely discontinuation of PFIF is important to lessen satiety, encourage hunger for solid foods, and help support age-appropriate progression with protein substitutes and food textures [24,25].

Social factors, such as maternal age, education, employment status, family structure (single-mother vs. two-parent households), and household income, influence infant feeding practices [28,29]. In our study, children from two-parent families or with mothers accomplishing higher education after secondary/high school were more likely to discontinue PFIF before 2 years of age, demonstrating the impact of family structure and maternal education. Prolonged bottle use offers satiety and is easier and more convenient than preparing complex, time-consuming low-protein meals. However, if the discontinuation of PFIF is delayed, it may hinder the development of healthy eating behaviors and limit the dietary diversity offered to young children. It is important for parents and healthcare professionals to plan and facilitate the timely and gradual transition from PFIF to age-appropriate protein substitutes. Healthcare professionals should also consider the demographic and socioeconomic factors influencing infant feeding practices to tailor effective guidance on PFIF discontinuation and transitioning to the next steps.

The extended use of night feeds was a concern. Although night feeds may offer a potential benefit by suppressing blood phenylalanine levels and improving morning metabolic control, there are many disadvantages associated with this practice. Several studies have reported that patients with PKU experience poor dental and periodontal conditions, along with a higher prevalence of dental caries compared to healthy controls [30,31,32,33]. Predisposing factors contributing to dental problems in PKU include inadequate oral hygiene, high carbohydrate intakes, frequent snacking habits, irregular dental check-ups, and the relatively lower priority placed on dental health due to the demands of managing the disorder [31]. Prolonged bottle use after 12 months of age is also an important risk factor [34,35]. Notably, all children who had known dental problems in our study (n = 5) were those who received PFIF after 2 years of age with night feeds later than 12 months, suggesting an important relationship. It is recommended to introduce a cup at 6 months and complete the transition from bottle to cup by 12 to 18 months [36]. Additionally, infants should not sleep with a bottle [37].

Another interesting finding was the prevalence of constipation symptoms during the first 6 months of life (44%), which improved with time, with only one patient experiencing constipation long term. Constipation is more common in formula-fed infants compared to breast-fed infants [38,39,40,41]. This finding may be attributed to differences in the lipid structure between breastmilk and infant formulae. Mehrotra et al. [42] reported that vegetable oil-based infant formulae were mainly associated with long-chain saturated fats esterified at sn-1 (stereospecific numbering) and sn-3 positions (breastmilk has fatty acids esterified at the sn-2 position) and are associated with the formation of calcium fatty-acid soaps, which in turn contributes to constipation. In healthy infants, several studies [43,44,45,46], but not all [47,48], have indicated that infants fed high sn-2 palmitate formula have softer stools, suggesting that reducing the palmitic acid content in infant formula or using synthetic triglycerides with palmitic acid primarily positioned at the sn-2 position could potentially improve stool consistency in infants.

This study provides valuable long-term follow-up data on growth, blood phenylalanine control, dietary intake, symptoms, and the experience with a PFIF in infants with PKU. However, several limitations should be acknowledged. The study design was retrospective and uncontrolled, relying on patient records, which may result in incomplete or underreported data. Generalizability to the PKU community is limited as the study was conducted at a single center in the UK. Anthropometric measurements were taken by different professionals, although equipment calibration followed hospital standards and professionals were trained in equipment use. Dietary intakes were collected by using 24 h food recall, which may not fully reflect the actual consumption, although the dietitians involved were experienced and familiar with patients’ dietary patterns. The use of complementary feeding and a second-stage protein substitute by some infants could have influenced the outcomes and weakened the strength of these findings. The assessment of infants’ experiences with the study formula relied on parental recollection, which introduces the potential for recall bias.

## 5. Conclusions

The PFIF given to infants with PKU in this study was well tolerated and accepted, resulting in satisfactory blood phenylalanine control and growth. However, ongoing monitoring and support with PFIF use and ensuring timely discontinuation and transition to age-appropriate weaning protein substitutes are important for optimizing PKU dietary management. The prescription of PFIF should be mainly replaced with a second-stage weaning protein substitute by 12 months and PFIF should be discontinued before 2 years of age, particularly if non-phenylalanine nitrogen requirements are met with a second-stage protein substitute. Future long-term studies should focus on long-term growth, weight gain, and developmental feeding outcomes in a larger cohort of infants with PKU.

## Figures and Tables

**Table 1:** Nutritional composition of the PFIF (PKU Start, Vitaflo International Ltd., Liverpool, UK) used in the study: comparison with standard infant formula (Aptamil^®^ First Infant milk, Nutricia International Pvt. Ltd., Trowbridge, Wiltshire, UK).

Nutritional Information (per 100 mL)	Units	PKU Start (Vitaflo)	Standard Infant Formula ^1^
Energy	kcal	68	66
	kj	287	276
Protein Equivalent	g	2.0	1.3
Carbohydrate	g	7.2	7.3
of which sugars	g	0.7	7.2
Fat	g	3.5	3.4
Saturated fat	g	0.9	1.1
Polyunsaturated fat	g	0.6	0.6
Linoleic acid	mg	536	N/A
Docosahexaenoic acid (DHA)	mg	14	16.5
Arachidonic acid (ARA)	mg	28	16.5
Alpha linolenic acid	mg	48	N/A
Monounsaturated fat	g	1.8	1.7
Vitamins			
Vitamin A	µg	65 (RE)	58
Vitamin D	µg	1.6	1.44
Vitamin E	mg αTE	0.99	1.3
Vitamin C	mg	8.6	9.2
Vitamin K	µg	5.6	5.5
Thiamin	mg	0.06	0.07
Riboflavin	mg	0.07	0.13
Niacin	mg	0.47	0.43
Vitamin B6	mg	0.06	0.05
Folate	µg	18	14
Vitamin B12	µg	0.17	0.16
Biotin	µg	2.7	1.9
Pantothenic acid	mg	0.35	0.53
Choline	mg	21	N/A
Minerals			
Sodium	mg	27	23
Potassium	mg	71	86
Chloride	mg	51	51
Calcium	mg	56	50
Phosphorus	mg	42	36
Magnesium	mg	6.3	5.4
Trace Elements			
Iron	mg	0.82	0.53
Copper	mg	0.06	0.05
Zinc	mg	0.49	0.48
Manganese	mg	0.04	0.003
Iodine	µg	15	13
Molybdenum	µg	2.3	N/A
Selenium	µg	3.0	2.5
Chromium	µg	2.0	N/A
Amino acids			
Essential amino acids			
Histidine	mg	80	34
Isoleucine	mg	150	75
Leucine	mg	230	136
Lysine	mg	150	119
Methionine	mg	40	33
Phenylalanine	mg	0	55
Threonine	mg	150	64
Tryptophan	mg	50	22
Tyrosine ^2^	mg	220	56
Valine	mg	170	81
Non-essential amino acids			
Alanine	mg	80	55
Arginine	mg	140	40
Aspartic acid	mg	220	123 ^3^
Cystine	mg	50	21
Glutamine	mg	160	282 ^4^
Glycine	mg	210	25
Proline	mg	150	116
Serine	mg	100	76

^1^ (Aptamil^®^ First Infant milk, Nutricia International Pvt. Ltd., Trowbridge, Wiltshire, UK). ^2^ Conditionally essential amino acid in PKU. ^3^ L-Aspartic Acid + L-Asparagine. ^4^ L-Glutamic acid + L-Glutamine. Abbreviations: kcal, kilocalorie; kj, kilojoule; g, gram; mg, milligram; µg, microgram; RE, retinol equivalents; TE, tocopherol equivalents; N/A, not applicable.

**Table 2:** Median weight-for-age, length-for-age, and BMI-for-age z-scores by age groups.

Age	Weight-for-Age z-Scores		Length-for-Age z-Scores		BMI-for-Age z-Scores	
	n	Median (Q1–Q3)	n	Median (Q1–Q3)	n	Median (Q1–Q3)
0 to 6 months	224	−0.3 (−1.3–0.4)	67	−0.1 (−1.0–0.5)	67	−0.4 (−1.1–0.4)
7 to 12 months	91	0.7 (0.0–1.3)	38	0.3 (−0.5–1.2)	37	1.0 (0.4–1.2)
13 to 24 months	108	0.7 (0.1–1.7)	56	0.0 (−0.7–1.1)	55	1.6 (0.5–2.0)

Abbreviations: Q1, first quartile; Q3, third quartile.

**Table 3:** Actual dietary intake by age.

	6 Months (n = 21) ^1^ Median (Q1–Q3)	12 Months (n = 24) ^1^ Median (Q1–Q3)	24 Months (n = 25) Median (Q1–Q3)
Energy			
kcal/day	671 (622–680)	935 (834–1056)	1000 (932–1099)
EAR %	105 (98–125)	126 (114–141)	106 (94–114)
Carbohydrate			
g/day	77 (73–88)	117 (102–140)	138 (123–153)
g/kg/day	11 (10–12)	11 (10–14)	10 (9–12)
% of energy	46 (45–47)	50 (48–54)	55 (51–59)
Fat			
g/day	30 (26–36)	33 (28–39)	31 (27–36)
g/kg/day	4.1 (3.6–5.1)	3.4 (2.6–4.2)	2.3 (2.1–2.9)
% of energy	40 (39–42)	33 (30–36)	27 (25–34)
Total protein			
g/day	24 (22–25)	39 (33–42)	42 (40–45)
g/kg/day	3.3 (3.0–3.5)	3.8 (3.4–4.1)	3.3 (3.0–3.6)
% of energy	14 (12–15)	16 (14–18)	16 (16–19)
Natural protein			
g/day	4 (4–5)	5 (5–6)	6 (5–6)
g/kg/day	0.6 (0.5–0.6)	0.5 (0.4–0.7)	0.4 (0.4–0.5)
% of energy	2.5 (1.9–2.8)	2.1 (2.0–2.7)	2.2 (1.8–2.6)
% of total protein	19 (15–20)	14 (11–18)	13 (11–16)
Total protein equivalent from infant and weaning protein substitute			
g/day	19 (18–22)	33 (27–36)	37 (34–41)
g/kg/day	2.7 (2.4–3.0)	3.3 (2.9–3.6)	2.8 (2.6–3.2)
% of energy	12 (10–12)	14 (12–16)	14 (13–16)
% of total protein	81 (79–85)	86 (83—89)	87 (84–89)
Recommended intakes (g/kg/day) [1]	2.0–3.0	2.0–3.0	1.5–2.0
Contribution from PFIF			
protein equivalent (g/day)	13 (10–16)	12 (8–15)	0 (0–8)
protein equivalent (g/kg/day)	1.8 (1.5–2.2)	1.2 (0.8–1.6)	0.0 (0.0–0.6)
% of energy	66 (60–72)	40 (31–50)	0 (0–22)
% of total protein	57 (48–66)	31 (19–41)	0 (0–17)
Contribution from weaning PS			
protein equivalent (g/day)	6 (4–8)	22 (15–26)	32 (30–37)
protein equivalent (g/kg/day)	0.8 (0.6–1.1)	2.1 (1.5–2.4)	2.7 (2.3–2.9)
% of energy	6 (5–11)	19 (16–25)	29 (24–33)
% of total protein	22 (18–33)	55 (44–64)	83 (73–86)

^1^ Breast-fed infants were excluded from the dietary analysis. Abbreviations: g, gram; kg, kilogram; kcal, kilocalorie; PS, protein substitute; PFIF, phenylalanine-free infant amino acid formula; EAR, estimated average requirements; Q1, first quartile; Q3, third quartile.

**Table 4:** Differences in blood phenylalanine by PFIF discontinuation.

Variable	PFIF Discontinued < 24 Months of Age (n = 16)		PFIF Discontinued ≥ 24 Months of Age (n = 9)		*p*
	n	Mean ± SD	n	Mean ± SD	
Blood phenylalanine (µmol/L)					
0 to 6 months	743	144 ± 123	422	159 ± 121	<0.05 *
7 to 12 months	624	172 ± 117	346	180 ± 131	>0.05
13 to 24 months	855	205 ± 136	507	178 ± 139	<0.05 *

* *p*-value significant at *p* < 0.05. Abbreviations: SD, Standard deviation.

**Table 5:** Differences in weight, length, and BMI z-scores by gender.

Variable	Male (n = 12)		Female (n = 13)		*p*
	n	Mean ± SD	n	Mean ± SD	
Weight-for-age z-score					
0 to 6 months	94	−0.7 + 1.3	130	−0.3 + 1.1	<0.05 *
7 to 12 months	44	0.5 + 1.4	47	0.4 + 0.9	>0.05
13 to 24 months	48	0.4 + 1.3	60	0.9 + 0.9	<0.05 *
Length-for-age z-score					
0 to 6 months	30	−0.2 + 1.1	37	−0.1 + 1.2	>0.05
7 to 12 months	20	0.6 + 1.4	18	0.0 + 1.1	>0.05
13 to 24 months	25	0.0 + 1.4	31	−0.1 + 1.2	>0.05
BMI-for-age z-score					
0 to 6 months	30	−0.3 + 1.0	37	−0.4 + 1.0	>0.05
7 to 12 months	20	0.9 + 1.0	17	0.3 + 1.1	>0.05
13 to 24 months	25	0.9 + 0.9	30	1.4 + 1.1	>0.05

* *p*-value significant at *p* < 0.05. Abbreviations: SD, Standard deviation; BMI, Body mass index.

**Table 6:** Differences in weight, length, and BMI z-scores by PFIF discontinuation.

Variable	PFIF Discontinued < 24 Months of Age (n = 16)		PFIF Discontinued ≥ 24 Months of Age (n = 9)		*p*
	Number of Assessments	Mean ± SD	Number of Assessments	Mean ± SD	
Weight-for-age z-score					
0 to 6 months	123	−0.1 ± 1.1	101	−0.9 ± 1.1	<0.05 *
7 to 12 months	53	0.5 ± 1.2	38	0.4 ± 1.1	>0.05
13 to 24 months	68	0.4 ± 1.1	40	1.1 ± 1.0	<0.05 *
Length-for-age z-score					
0 to 6 months	34	0.0 ± 1.1	33	−0.4 ± 1.2	>0.05
7 to 12 months	24	0.3 ± 1.2	14	0.4 ±1.5	>0.05
13 to 24 months	35	−0.1 ± 1.2	21	0.1 ± 1.5	>0.05
BMI-for-age z-score					
0 to 6 months	34	−0.1 ± 1.1	33	−0.5 ± 1.3	>0.05
7 to 12 months	23	0.5 ± 1.3	14	0.9 ± 0.5	>0.05
13 to 24 months	35	1.0 ± 1.0	20	1.6 ± 0.9	<0.05 *

* *p*-value significant at *p* < 0.05. Abbreviations: SD, Standard deviation; BMI, Body mass index; PFIF, phenylalanine-free infant amino acid formula.

**Table 7:** Differences in energy and macronutrient intake by PFIF discontinuation.

Variable	PFIF Discontinued < 24 Months of Age (n = 16)	PFIF Discontinued ≥ 24 Months of Age (n = 9)	*p*
	Mean ± SD	Mean ± SD	
Energy (kcal/day)			
6 months	653 ± 86	734 ± 101	>0.05
12 months	899 ± 145	1013 ± 139	>0.05
24 months	966 ± 79	1148 ± 192	<0.05 *
EAR % for energy			
6 months	103 ± 13	117 ± 17	>0.05
12 months	121 ± 19	138 ± 21	>0.05
24 months	100 ± 10	120 ± 22	<0.05 *
Carbohydrate (g/day)			
6 months	76 ± 8	83 ± 10	>0.05
12 months	117 ± 25	129 ± 23	>0.05
24 months	136 ± 20	147 ± 24	>0.05
Carbohydrate (g/kg/day)			
6 months	10.5 ± 1.4	11.3 ± 1.8	>0.05
12 months	11.8 ± 3.3	13.8 ± 3.6	>0.05
24 months	10.6 ± 2.2	11.4 ± 2.2	>0.05
Fat (g/day)			
6 months	29 ± 5	34 ± 6	>0.05
12 months	30 ± 7	39 ± 7	<0.05 *
24 months	28 ± 5	41 ± 13	<0.05 *
Fat (g/kg/day)			
6 months	4.0 ± 0.8	4.6 ± 0.9	>0.05
12 months	3.1 ± 0.8	4.2 ± 1.1	<0.05 *
24 months	2.2 ± 0.5	3.2 ± 1.1	<0.05 *
Total protein (g/day)			
6 months	23 + 4	24 + 2	>0.05
12 months	39 ± 5	35 ± 5	>0.05
24 months	42 + 7	45 + 4	>0.05
Total protein (g/kg/day)			
6 months	3.2 + 0.6	3.3 + 0.3	>0.05
12 months	3.9 ± 0.6	3.7 ± 0.5	>0.05
24 months	3.3 + 0.5	3.5 + 0.5	>0.05
Natural protein (g/kg/day)			
6 months	0.5 + 0.1	0.6 + 0.2	>0.05
12 months	0.5 ± 0.1	0.6 ± 0.1	>0.05
24 months	0.5 + 0.1	0.4 + 0.1	>0.05
Protein Equivalent (g/kg/day)			
6 months	2.7 + 0.6	2.7 + 0.4	>0.05
12 months	3.4 ± 0.5	3.1 ± 0.5	>0.05
24 months	2.8 + 0.4	3.1 + 0.5	>0.05

Data from four infants at 6 months and one infant at 12 months were excluded due to their ongoing breastfeeding. * *p*-value significant at *p* < 0.05. Abbreviations: SD, Standard deviation; PFIF, phenylalanine-free infant amino acid formula; EAR, estimated average requirements; g, gram; kg, kilogram; kcal, kilocalorie.

**Table 8:** Simple (n) and relative (%) frequency of children who discontinued PFIF ≥ 24 months of age according to demographic and socioeconomic factors.

Variables	Total	PFIF Discontinued ≥ 24 Months of Age	*p*
	n	n (%)	
Sex			>0.05
Male	12	3 (25)	
Female	13	6 (46)	
Siblings			>0.05
0	5	2 (40)	
1–2	13	3 (23)	
3–4	7	4 (57)	
Family size (parents included)			>0.05
2–3	5	2 (40)	
4–5	14	5 (36)	
6–7	6	2 (33)	
Parental marital status (n, %)			<0.05 *
Two parents	18	4 (22)	
Single parent ^1^	7	5 (71)	
Maternal education			>0.05
Up to 16 years old only	13	7 (54)	
Diploma/Degree	12	2 (17)	

^1^ Widowed, divorced, or separated. * *p*-value significant at *p* < 0.05. Abbreviations: PFIF, phenylalanine-free infant amino acid formula.

## Data Availability

The data presented in this study are available upon request from the corresponding author. The data are not publicly available due to privacy reasons.
